# A novel pathogenic species of genus *Stenotrophomonas*: *Stenotrophomonas pigmentata* sp. nov

**DOI:** 10.3389/fcimb.2024.1410385

**Published:** 2024-06-05

**Authors:** Yue Li, Zelin Yu, Xueting Fan, Da Xu, Haican Liu, Xiuqin Zhao, Ruibai Wang

**Affiliations:** National Institute for Communicable Disease Control and Prevention, Chinese Centre for Disease Control and Prevention, Beijing, China

**Keywords:** *Stenotrophomonas*, tuberculosis, pathogen, multiple-drug resistance, pigment

## Abstract

**Introduction:**

*Stenotrophomonas* is a prominent genus owing to its dual nature. Species of this genus have many applications in industry and agriculture as plant growth-promoting rhizobacteria and microbial biological control agents, whereas species such as *Stenotrophomonas maltophilia* are considered one of the leading gram-negative multi-drug-resistant bacterial pathogens because of their high contribution to the increase in crude mortality and significant clinical challenge. Pathogenic *Stenotrophomonas* species and most clinical isolates belong to the *Stenotrophomonas maltophilia* complex (SMc). However, a strain highly homologous to *S. terrae* was isolated from a patient with pulmonary tuberculosis (TB), which aroused our interest, as *S. terrae* belongs to a relatively distant clade from SMc and there have been no human association reports.

**Methods:**

The pathogenicity, immunological and biochemical characteristics of 610A2^T^ were systematically evaluated.

**Results:**

610A2^T^ is a new species of genus *Stenotrophomonas*, which is named as *Stenotrophomonas pigmentata* sp. nov. for its obvious brown water-soluble pigment. 610A2^T^ is pathogenic and caused significant weight loss, pulmonary congestion, and blood transmission in mice because it has multiple virulence factors, haemolysis, and strong biofilm formation abilities. In addition, the cytokine response induced by this strain was similar to that observed in patients with TB, and the strain was resistant to half of the anti-TB drugs.

**Conclusions:**

The pathogenicity of 610A2^T^ may not be weaker than that of *S. maltophilia*. Its isolation extended the opportunistic pathogenic species to all 3 major clades of the genus *Stenotrophomonas*, indicating that the clinical importance of species of *Stenotrophomonas* other than *S. maltophilia* and potential risks to biological safety associated with the use of *Stenotrophomonas* require more attention.

## 1 Introduction

*Stenotrophomonas* is a member of the family *Xanthomonadaceae*. Because of advancements in molecular biology and identification technology, the number of species in this genus has increased rapidly in recent years. Based on our interpretation of the current available NCBI taxonomy, from the first species, *Stenotrophomonas maltophilia*, isolated in 1885 to 2011, 16 strains have been isolated within 117 years, and 12 species were identified within 9 years from 2016 to 2024. *Stenotrophomonas* is ubiquitous and associated with a wide range of habitats, including humans, animals, plant hosts, and extreme environments. Most members of *Stenotrophomonas* are particularly known for producing protective osmotic substances and are considered as plant rhizosphere growth-promoting bacteria. They also have valuable applications in the fields of agriculture and industry as emerging sources of biodegradation and substitutes for synthetic fungicides (Kumar et al., 2023). *Stenotrophomonas* strains can interact with other microorganisms on plant surfaces and in the soil through biofilms, inhibit plant pathogenic fungi and viruses, and alter the composition of rhizosphere microorganisms through extracellular enzyme decomposition and competition for iron. By inducing the enrichment of plant hormones, such as jasmonic acid, and upregulating the transcription level of jasmonic acid-sensitive genes, *Stenotrophomonas* strains help plants defend against various crop pests, such as *Spodoptera litura*. Their function in promoting plant growth is through the production of auxin and hydrogen cyanide; dissolution of phosphorus and potassium salts; nitrogen fixation; enzymatic degradation of soil odour, explosive pollutants, keratin, macrocyclic hydrocarbons, nitrophenols, and other substances; removal of various chemical pesticides, insecticides, and environmental pollutants; and effective bioremediation of agricultural soil without harming the ecosystem (Kumar et al., 2023).

A few species of this genus are pathogenic to humans and are represented by *S. maltophilia*. As a globally emerging organism, *S. maltophilia* is recognised for its multi-drug resistance (Gil-Gil et al., 2020), ability to cause various infections in the human body, and high contribution to the increase in crude mortality (Tan et al., 2014). Based on global clinical data, the attributed mortality rates for pulmonary infection and bacteraemia caused by this bacterium are as high as 30% and 65%, respectively (Zhou et al., 2013). *S. maltophilia* is listed by the World Health Organization as one of the leading gram-negative, multi-drug-resistant bacterial pathogens in hospitals (Gröschel et al., 2020) and poses a great clinical challenge (Mojica et al., 2022; Tamma et al., 2022).

Because of their phenotypic and genotypic diversity, the taxonomic status of the genus *Stenotrophomonas* and *S. maltophilia* has changed several times. Besides the species in the *Stenotrophomonas maltophilia* complex (SMc), *S. maltophilia* (Chang et al., 2015; Patil et al., 2018; Bansal et al., 2021; Brooke, 2021) and *S. sepilia* (Gautam et al., 2021), only *S. pavanii* (Iebba et al., 2018), *S. acidaminiphila* (Ježek et al., 2020; Zhang et al., 2022) and *S. rhizophila* (Chao et al., 2022) have been isolated from human samples. The clinically important species *S. africana* (Coenye et al., 2004) is classified as a synonym of *S. maltophilia*, whereas *S. pavanii* has been removed from SMc (Rouf et al., 2011; Patil et al., 2018; Maturana and Cardenas, 2021; Deng et al., 2022). Most of the clinical isolates belong to the 23 monophyletic lineages of SMc (Gröschel et al., 2020), and only *S. acidaminiphila* and *S. rhizophila* have no close genetic relationship to *S. maltophilia*.

In our previous small surveillance conducted in a district tuberculosis (TB) hospital in Beijing, we found that *Stenotrophomonas* was a major co-occurring species of *Mycobacterium tuberculosis*, with a crude isolation rate of 6.74%. *Stenotrophomonas* may be an important opportunistic pathogen for patients with TB, similar to that for patients with cystic fibrosis (Li et al., 2023). On the basis of 16S rDNA sequencing, 2 out of 9 *Stenotrophomon*as isolates did not belong to *S. maltophilia*, and one had the highest identity with *S. terrae* strain AFS037341 (99.24%) and *S. humi* strain AFS068096 (98.96%). These similarity values were more than 98.65%, which has been previously used as the threshold for differentiating *Stenotrophomonas* species (Deng et al., 2022). To the best of our knowledge, *S. terrae* and its closely related species have not been isolated from human resources, which sparked our interest in the pathogenicity of these strains. However, after PacBio whole-genome sequencing, the average nucleotide identity (ANI) between the strain 610A2^T^, which was highest similar to *S. terrae*, and all existing species of the genus *Stenotrophomonas* was less than 88%. Therefore, in addition to animal experiments and pathogenicity assessments, we conducted a systematic evaluation of 610A2^T^ according to the description required for new taxa.

## 2 Materials and methods

### 2.1 Sources of isolates

Strain 610A2^T^ was isolated from a 26-year-old male patient with pulmonary TB under treatment at the tuberculosis clinic of the Chaoyang District Center for Disease Control and Prevention (39.89 N, 116.40 E) in our previous surveillance under ethics approval No. ICDC-2022010 (Li et al., 2023). It was first identified as *Stenotrophomonas* by amplification and sequencing with 16S rDNA universal primers for bacteria (16S-U: 5′AGA GTT TGA TCM TGG CTC AG 3′ and/L: 5′ CCG TCA ATT CMT TTR AGT TT 3′).

### 2.2 Whole-genome sequencing and phylogenetic analysis

Genome of 610A2^T^ was sequenced using the PacBio sequel II and DNBSEQ platform at the Beijing Genomics Institute (BGI, Shenzhen, China). Four SMRT cell zero-mode waveguide arrays for sequencing were used to generate the sub-read set. The PacBio subreads (length < 1 kb) were removed. Canu and GATK (https://www.broadinstitute.org/gatk/) were used for self-correction and single-base corrections, to improve the accuracy of the genome sequences.

Gene prediction was performed using glimmer3 with Hidden Markov models (http://www.cbcb.umd.edu/software/glimmer/). Other genomic elements, such as RNA, tandem repeats, genomic regions, small satellite DNA, and microsatellite DNA, were identified using tRNAscan SE, RNAmmer, Tandem Repeat Finder (http://tandem.bu.edu/trf/trf.html), Genomic Island Suite of Tools (http://www5.esu.edu/cpsc/bioinfo/software/GIST/), PHAge Search Tool (PHAST, http://phast.wishartlab.com), and CRISPRFinder. Eleven databases, namely, KEGG (Kyoto Encyclopedia of Genes and Genomes, http://www.kegg.jp), COG (Clusters of Orthologous Groups, http://www.ncbi.nlm.nih.gov), NR (Non-Redundant Protein Database databases, ftp://ftp.ncbi.nih.gov/blast/db/FASTA/nr.gz), Swiss-Prot (http://ngdc.cncb.ac.cn), GO (Gene Ontology, http://www.geneontology.org), TrEMBL, EggNOG (http://eggnog.embl.de), VFDB (Virulence Factors of Pathogenic Bacteria, http://www.mgc.ac.cn), ARDB (Antibiotic Resistance Genes Database, http://www.argodb.org/), CAZy (Carbohydrate-Active enZYmes Database, http://www.cazy.org), and T3SS (Type III secretion system effector protein, EggNOG database 1.0.1, http://effectivedb.org), were used for general function annotation and pathogenicity/drug resistance analysis.

Complete 16S rDNA gene sequences were extracted from the sequenced genome and reference genomes of 26 species of the genus *Stenotrophomonas*, except for *S. detusculanense* and *S. indologenes*, which do not have publicly available genome sequences. ClustalX was used for multiple sequence alignments, and a bootstrap neighbour-joining (NJ) phylogenetic tree was constructed using Treeview (1.6.6) with 1000 replicas. ANI was estimated using the ANI calculator (http://enve-omics.ce.gatech.edu/ani/). Syntenies were performed using MUMmer and BLAST. Core/Pan genes were clustered by CD-HIT rapid clustering of similar proteins software with a threshold of 50% pairwise identity and 0.7 length difference cutoff in amino acid. Hcluster_sg software was used to perform gene family clustering, and a phylogenetic tree was constructed using TreeBeST with the NJ method.

### 2.3 Examination of phenotypic and biochemical characteristics

The strain’s growth ability was tested on 4 media, tryptone soy (TSA; Difco), Luria-Bertani (LB; Difco), Columbia blood agar (OXOID), and Mueller-Hinton (MH; Difco), at 37 °C for 24–72 h. Growth at different temperatures (4, 15, 20, 25, 28, 35, 37, 40, and 45 °C), pH (pH 4–11, at intervals of 1 pH unit, 37 °C) and NaCl concentrations (0–10%, w/v, in intervals of 1%) was also evaluated using LB broth and agar at 37 °C. Cellular motility was monitored using 0.75% semi solid agar medium, and morphological features were observed using a transmission electron microscope (HT7700; Hitachi, Japan). Utilisation of carbon sources and enzyme production were tested with API 20NE (48 h, 28 °C), API ZYM (4 h, 28 °C), and API 50 CH (inoculated with AUX medium, 48 h, 28 °C) (20050, 50300, 25200; BioMerier France, France), according to the manufacturer’s instructions. Cellular fatty acids and matrix-assisted laser desorption/ionization time-of-flight mass spectrometry (MALDI-TOF) were analysed using the Sherlock Microbial Identification System (MIDI) with the MIDI Sherlock software program (version 6.3), RTSBA6 (6.21) library, and Autoflex speed TOF/TOF (Bruker Daltonics GmbH, Germany), according to the manufacturers’ instructions.

### 2.4 Antibiotic susceptibility testing

Two kinds of AST plates, the customised AST plate for Chinese Pathogen Identification Net (CHNENF, Trek Diagnostic Systems Ltd, West Sussex, United Kingdom) and the Sensititre^™^ MYCOTB minimum inhibitory concentration (MIC) plate (Trek Diagnostic Systems, Cleveland, OH, USA), were used to determine the strain’s sensitivity to 17 drugs commonly used for gram-negative strains and 12 anti-TB drugs. MICs were determined according to the standards of the Clinical and Laboratory Standard Institute (CLSI, 2018) and manufacturer’s instructions.

### 2.5 Biofilm formation

Biofilm formation was determined using crystal violet staining. Fresh bacterial suspension was prepared and adjusted to 0.5 McFarland. After 1:30-fold dilution with tryptone soy broth (TSB; Difco), 150 μL/well suspension was added to a 96-well flat-bottom microtitre plate, and the plate was incubated at 37 °C. At each time point, bacterial turbidity (OD_630 nm_) was measured using an enzyme-linked immunosorbent assay (ELISA) reader (Bio-Rad 680; Bio-Rad Laboratories, Japan). The cultures were discarded, and the wells were washed 3 times with PBS (pH 7.3) to remove planktonic cells. The biofilms were stained with 200 μL/well of 0.1% crystal violet for 15 min. The wells were washed again, and 200 μL of 99% ethanol was added and OD_403 nm_ was measured after 15 min. The biofilm index was calculated as OD_403 nm_/OD_630 nm_. Six clinically isolated *S. maltophilia* strains, including strain 11066 (GDMCC 1.4335), were used as controls.

### 2.6 Animal infection experiment

Animal experiments were conducted with the approval of the Laboratory Animal Welfare & Ethics Committee of the National Institute for Communicable Disease Control and Prevention (Issue number 2023-021).

Using a random number method, female Kunming mice aged 6–7 weeks and weighing 30–35 g were randomly divided into infection and control groups. Five mice in each infection group were intranasally inoculated with 50 μL of 1.5 × 10^8^ CFU/mL of tested strains, and mice in the control groups were inoculated with PBS. At 4 h, 12 h, 1 day, 2 days, 3 days, 5 days, and 7 days post-infection, the mice were sacrificed and the lung and spleen tissues were collected aseptically.

The left lobes of the lungs were fixed in 10% neutral formalin for 24 h, followed by tissue processing and paraffin embedding. The paraffin blocks were sectioned at 2 µm, stained with haematoxylin and eosin, and blindly examined under a microscope by an expert in the field of laboratory animal pathology. Pulmonary clearance and occurrence of disseminated infection were monitored via quantitative bacteriology of the lung and spleen homogenates, respectively. Briefly, tissues were homogenised (1000 rpm) on ice in 1 mL of sterile normal saline by using a homogeniser (RZ-GR96A; Beijing Guoke Rongzhi Biotechnology Co., Ltd., China). Then, 100 μL of homogenates and 10-fold serial dilutions were inoculated on LB agar. The number of colonies was counted 24–48 h after incubation at 37°C. Bacterial colony counts were normalised according to the wet tissue weight and calculated as CFU/g. The levels of murine tumour necrosis factor alpha (TNF-α), granulocyte-macrophage colony-stimulating factor (GM-CSF), gamma interferon (IFN-γ), interleukin (IL)-2, IL-4, IL-6, IL-10, IL-12p70, and IL-17A were quantified using Luminex technology (R&D Systems, Minneapolis, MN, USA) and reagents (12002798; BIO-RAD, USA).

### 2.7 Statistical analysis

For comparison of multiple groups, one-way ANOVA was performed using GraphPad Prism version 9.3.1 for Windows (GraphPad Software, San Diego, California, USA).

## 3 Results

### 3.1 Genomic characteristics and classification into the genus *Stenotrophomonas*

The total length of 610A2^T^ complete genome was 4,681,496 bp, and GC content was 63.29% with an N50 length of 11,086 bp. A total of 4,070 CDSs, 70 tRNAs, 6 5S rRNAs, 4 16S rRNAs, 4 23S rRNAs, 37 sRNAs, 353 tandem repeats, 175 minisatellite DNAs, and 90 microsatellite DNAs were annotated.

**Figure 1:**
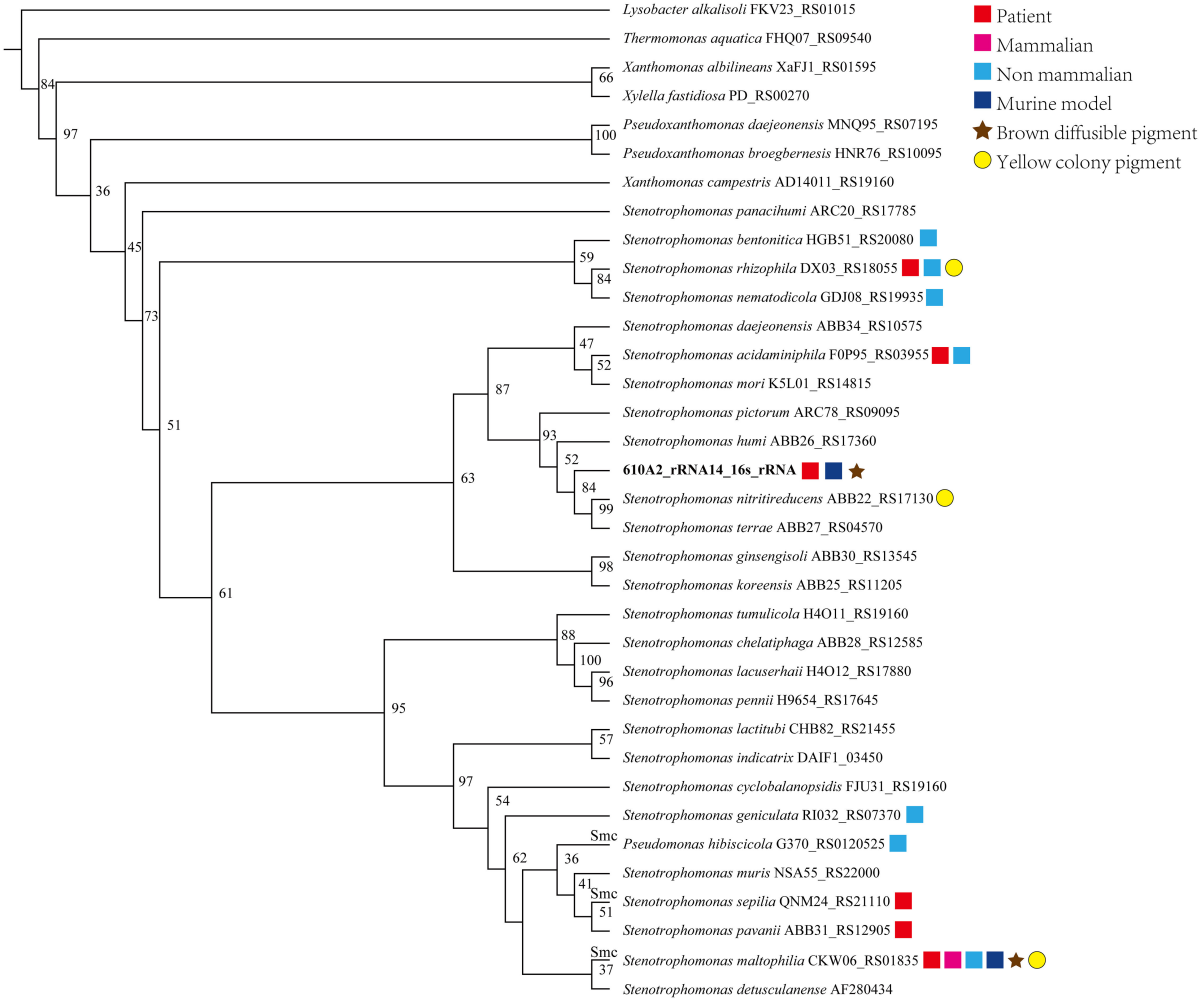
Phylogenetic tree created on the basis of complete 16S rDNA gene sequences of 610A2^T^ and reference genomes of 26 species *Stenotrophomonas*. ClustalX was used for multiple sequence alignments, and a bootstrap neighbour-joining (NJ) phylogenetic tree was constructed using Treeview (1.6.6) with 1000 replicas. Species isolated from patients or animals or tested on a murine model are marked with symbols following their species name and 16S rDNA locus_tag.

**Figure 2:**
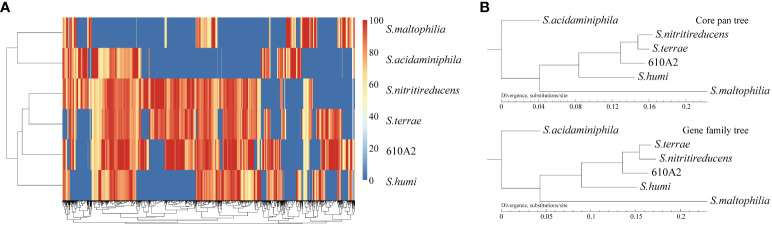
Gene heat map **(A)** and phylogenetic trees based on corePan and genefamily1 results **(B)**.

In the phylogenetic tree based on the complete 16S rDNA gene (1545 bp), 610A2^T^ was clustered in a clade including *S. terrae*, *S. nitritireducens*, *S. humi*, and *S. pictorum* and formed a subclade between *S. terrae* and *S. humi* (**Figure 1**). Dispensable gene heat maps and phylogenetic trees based on corePan and genefamily1 results showed completely consistent positioning (**Figures 2A, B**). When compared with the 26 *Stenotrophomonas* type species, 610A2^T^ displayed lower ANI values ranging from 87.53 to 80.22, less than 95%, the generally accepted threshold for species (Maturana and Cardenas, 2021; Deng et al., 2022). Synteny analyses at the nucleotide and amino acid levels were compared with 8 type strains: *S. humi* DSM 18929, *S. sepilia* SM16975, *S. geniculata* FLMAT1, *S. maltophilia* NCTC10257, *S. terrae* DSM 18941, *S. nitritireducens* DSM 12575, *S. rhizophila* DSM 14405, and *S. acidaminiphila* T0-18. *S. terrae* had the highest homology, with a gene median identity of 91.34% and map length rate of 63.327% (**Table 1**). These data suggest that 610A2^T^ represents a novel species within the genus *Stenotrophomonas*, and it is closely related to, but distinct from, *S. terrae*. The name *Stenotrophomonas pigmentata* sp. nov. is proposed for the distinct brown water-soluble pigment it produces.

**Table 1:** ANI values and synteny analyses at the nucleotide and amino acid levels between 610A2 and 8 type strains.

Species/Strain name	ANI (%)	Nucleotide levels			Amino acid levels					
		Map Num	Map Length	Rate (%)	Aligned	Target Percent (%)	Query Genes	Query Percent (%)	Identity Mean	Identity Median
*S. humi*	83.94	459	1033761	22.082	2965	72.85	3612	82.09	85.14	88.01
*S. sepilia*	81.05	188	265466	5.671	2535	62.29	4158	60.97	74.76	76.92
*S. geniculata*	81.26	196	302519	6.462	2570	63.14	4822	53.3	74.93	77.17
*S. maltophilia*	81.16	184	275081	5.876	2582	63.44	4007	64.44	74.93	77.155
*S. terrae*	87.53	2630	2964645	63.327	3201	78.65	3770	84.91	91.34	94.26
*S. nitritireducens*	87.47	2112	2787879	59.551	3126	76.81	3812	82	91.13	94.04
*S. rhizophila*	81.18	204	289657	6.187	2614	64.23	3938	66.38	75.46	77.9
*S. acidaminiphila*	83.08	343	686404	14.662	2598	63.83	3617	71.83	81.13	83.62

### 3.2 Pathogenicity in animal infection experiment

At the infection dose of 50 μL of 1.5 × 10^8^ CFU/mL, no mortality was observed in either the infection or control group. The mice infected with 610A2^T^ showed a weight loss of 3.9 ± 0.9%, 8.0 ± 0.7%, 6.4 ± 1.3%, 2.3 ± 1.2%, 0.8 ± 1.3% at 12 h, 1 day, 2 days, 3 days, and 5 days post-infection, respectively. On the seventh day, weight gain resumed. In addition, no significant differences were observed in the physical signs between the infection and control groups.

The bacterial load per tissue weight (g) in the lung measured via viable count was 1.1 ± 1.5 × 10^7^ CFU/mL·g at 4 h, 1.8 ± 3.6 × 10^5^ CFU/mL·g at 12 h, and 2.0 ± 4.0 × 10^3^ CFU/mL·g at 1 day. Bacteria could not be isolated from lung homogenates 2 days post-infection. 610A2^T^ was isolated only from the spleen homogenate of all infected mice within 4 h, with a viable bacterial count of 1.1 ± 2.2 × 10^3^ CFU/mL g, indicating that 610A2^T^ has strong invasive ability but the host can also quickly clear it.

**Figure 3:**
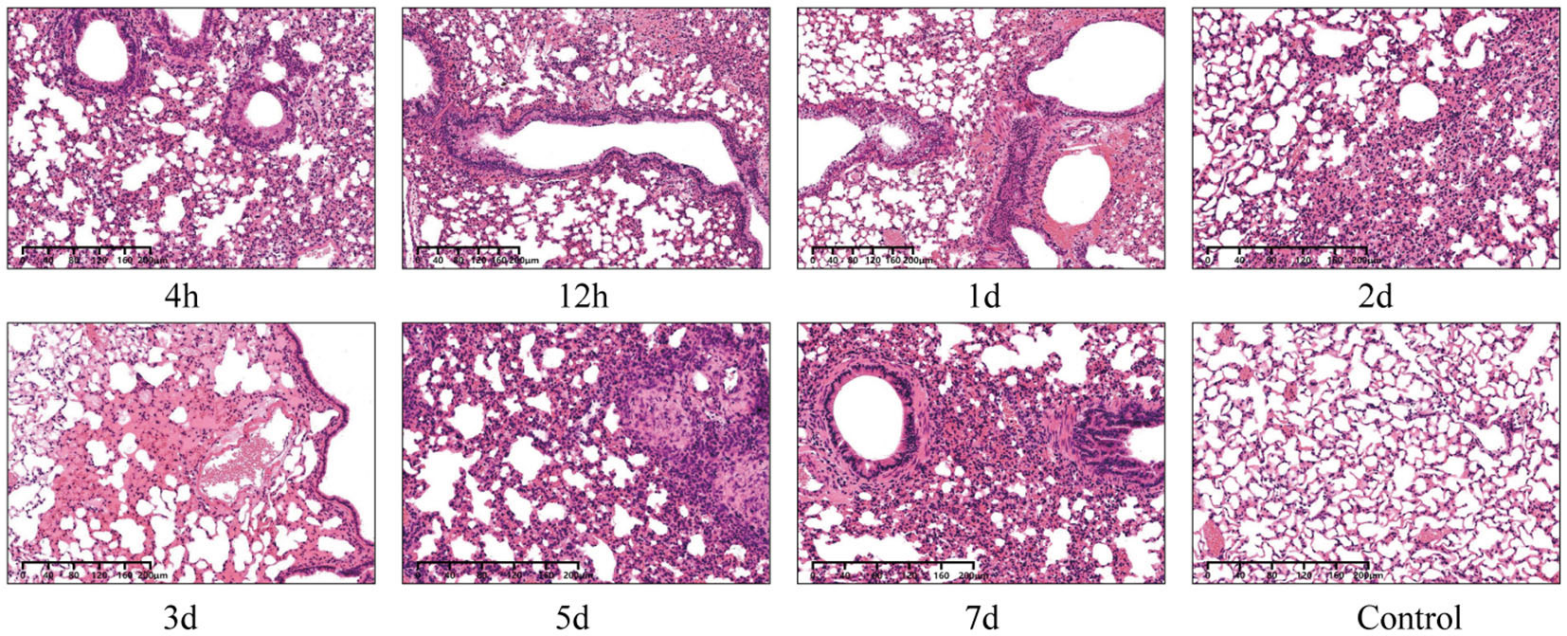
Microscopic images of lung histopathology of KM mice intranasally infected with 610A2^T^ at 7 timepoints (4 h, 12 h, 1 day, 2 days, 3 days, 5 days, and 7 days post-infection). The lung structure of the control group was normal, whereas infiltration of inflammatory cells, compensatory alveolar dilation, mucosal epithelial oedema, and even obvious bloody exudate was observed in the infected lung sections.

Histological examination showed that pulmonary damage in the infected group constantly included alveolar septa thickening, congestion, bleeding, inflammatory cell infiltration, compensatory alveolar dilation, mucosal epithelial oedema, and vacuolar degeneration (**Figure 3**). Although the viable count showed that the bacteria in the lungs had been cleared after 2 days, obvious bloody exudate and a large number of inflammatory cells were detected in the alveoli in 1–3 days, which is consistent with the symptoms for pulmonary congestion. After 7 days, the lung structure of the infected group was restored to normal, whereas that of the control group was normal.

**Figure 4:**
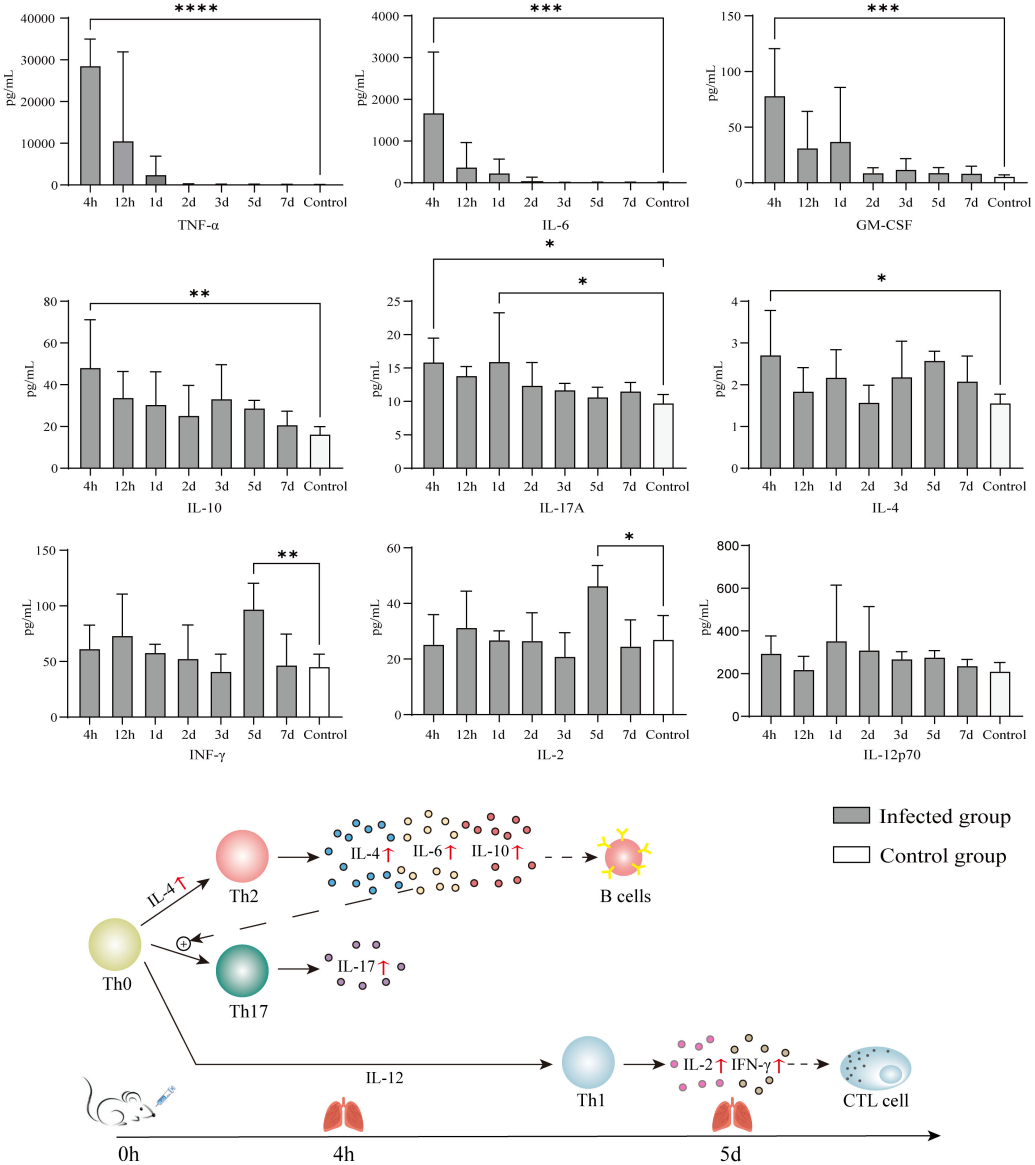
Immune response of KM mice intranasally infected with 610A2^T^. At 4 h, 12 h, 1 day, 2 days, 3 days, 5 days, and 7 days post-infection, the mice were sacrificed and 9 cytokines, TNF-α, GM-CSF, IFN-γ, IL-2, IL-4, IL-6, IL-10, IL-12p70, and IL-17A, in the lung homogenates were measured using Luminex. Values are mean ± SD (n = 10) obtained from 2 independent experiments. One-way ANOVA followed by Bonferroni’s multiple comparison post-test were used for statistical testing, and the groups with significant differences when compared with the control were marked with asterisks. * *P* < 0.03, ** *P* < 0.002, ***P < 0.0002, **** *P* < 0.0001.

Eight of the 9 quantified cytokines showed significant differences between infected and control groups (**Figure 4**). The means of TNF-α, GM-CSF, IL-4, IL-6, IL-10, and IL-17A at each timepoint were higher than those of the control group, but only the values at 4 h had statistical significance because of the variability caused by individual differences. IFN-γ and IL-2 showed a consistent trend of change, lagging behind other cytokines and showing significant differences from the control group on the 5th day; however, at this time point, TNF-α and IL-6 had recovered to levels no different from the control group.

### 3.3 Prediction of virulence factors

Functional annotation revealed that the 610A2^T^ genome contained 304 virulence factors of pathogenic bacteria in VFDB and 100 human disease pathway genes in KEGG database, of which 16 genes were related to bacterial infectious diseases. In details, 53 genes were related to flagellar biosynthesis, flagellar basal body protein, flagellar motor switching, and chemotaxis; 32 type IV pili genes; 3 outer membrane usher protein genes; 24 iron/haeme uptake and utilisation relative genes; and 1 biofilm-controlling response regulator. 610A2^T^ harbours 2 haemolysin-encoding genes, *hlyB* and *hlyIII*, as well as the haemolysin activation/secretion protein gene *fhaC*, a transcriptional regulator of haemolysin *slyA*, and 3 HlyD family secretion proteins, which are consistent with the haemolytic activities on Columbia blood agar containing sheep blood.

The annotation results showed that 610A2^T^ may possess 4 types of secretion systems, consisting of 3 T3SS genes, 5 T4SS effectors, 1 T5SS gene, 19 T6SS genes (*tssA*-*H*, *tssK*, *tssL*, and *tssM*), concentrated in 2 regions of the chromosome. In addition, 610A2^T^ harbours 9 lipopolysaccharide biosynthesis genes; 11 haeme biosynthesis and utilisation genes; 4 superoxide dismutase genes (Fe-Mn and Cu-Zn families); 1 superoxide oxidase gene; 3 glutathione peroxidase genes; 2 metalloendopeptidase OMA1; 1 melanin-producing gene cluster containing MarR transcriptional regulator, 4-hydroxyphenylpyruvate dioxygenase, and homogentisate 1,2-dioxygenase; 3 catalase genes; 12 alginate biosynthesis and regulation genes; 2 nitrate reductase genes; 1 isocitrate lyase gene; and 1 succinate dehydrogenase gene.

### 3.4 AST and related genes

Owing to the slow growth rate of 610A2^T^, its AST could not be tested using the BD Phonix-100 equipment (BD, Sparks, MD, USA) with BD Phonix™ NMIC 413 panels. Among the drugs detected with CHNENF and MYCOTB plates, 610A2^T^ was sensitive to 10 drugs: azithromycin (8 μg/mL), chloramphenicol (16 μg/mL), tetracycline (8 μg/mL), streptomycin (16 μg/mL), tigecycline (0.5 μg/mL), ofloxacin (1 μg/mL), moxifloxacin (0.06 μg/mL), and rifampin (0.12 μg/mL), with amikacin (<4 μg/mL) and colistin (<0.25 μg/mL) being the most sensitive. 610A2^T^ was resistant to ertapenem (8 μg/mL) and rifabutin (0.5 μg/mL). Fifteen drugs exceeded the maximum detection concentrations of the assay: cycloserine (>256 μg/mL), kanamycin (>40 μg/mL), para-aminosalicylic acid (>64 μg/mL), ethionamide (>40 μg/mL), isoniazid (>4 μg/mL), ethambutol (>32 μg/mL), cefotaxime (>16 μg/mL), ceftazidime (>32 μg/mL), ceftazidime/avibactam (>16/4 μg/mL), meropenem (>2 μg/mL), ampicillin/sulbactam (>32/16 μg/mL), ciprofloxacin (> 0.12 μg/mL), nalidixic acid (> 32 μg/mL), ampicillin (> 32 μg/mL), and trimethoprim-sulfamethoxazole (TMP-SMX, > 8/152 μg/mL).

Comparison against the ARDB, CARD, and KEGG databases revealed that 610A2^T^ carried 41 antibiotic-resistant relative genes on its genome, including 12 genes, *aac6-I, acrb, adeb, ceob, emre, macb, mexc, mexe, oprn, smed, smee*, and *ykkd*, coding for multi-drug resistance efflux pump for chloramphenicol, aminoglycoside, fluoroquinolone, macrolide, and tetracycline resistance; extended-spectrum beta-lactamase (ESBL) gene *blaCTX-M*, subclass B3 metallo-β-lactamase gene *blaB*, and *pbp1a* and *pbp2b* for cephalosporin, penicillin, and β-lactam resistance; *rosA* and *rosB* for fosmidomycin resistance; as well as some special resistant genes, *bacA* for bacitracin, *ksgA* for kasugamycin, *dfrA-*26 for trimethoprim, and *vanA* for vancomycin and teicoplanin.

### 3.5 Systematic evaluation of physiological and chemotaxonomic properties

**Figure 5:**
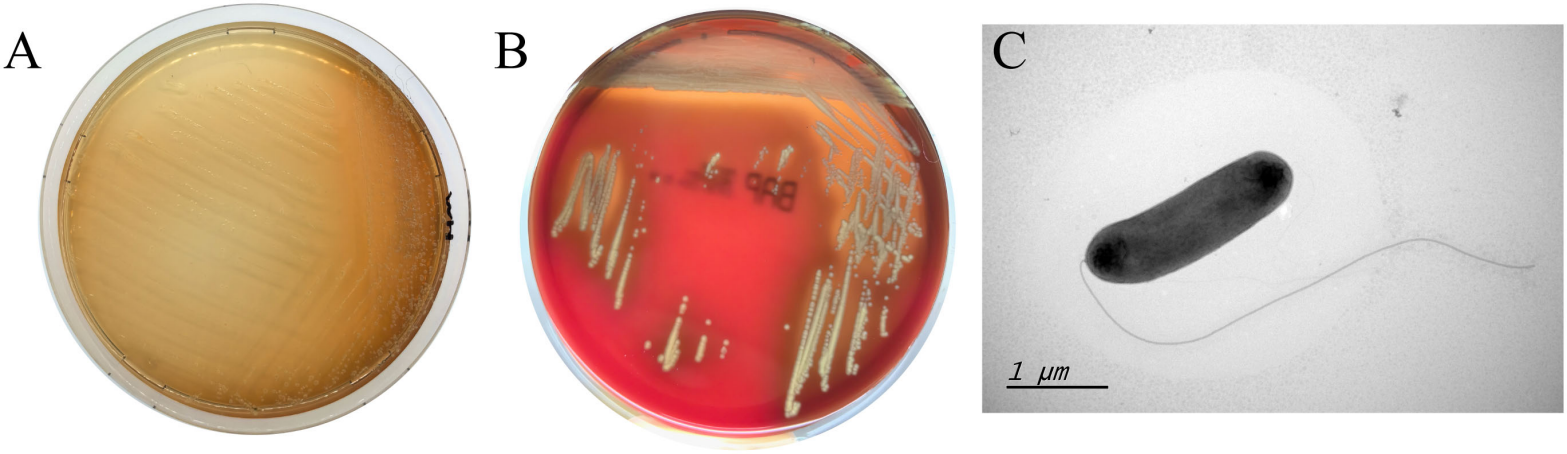
Clones of 610A2^T^ on MH medium **(A)**, columbia blood agar **(B)** and its transmission electron microscopy image **(C)**. Obvious brown water-soluble pigment and haemolytic ring can be observed on MH medium and blood agar, respectively. The bacterial body of 610A2^T^ is about 1.5–2 × 0.5 μM with a single extreme flagella.

610A2^T^ grew in all 4 tested media. The colonies on nutrient agar were translucent, smooth, and moist, with brown water-soluble pigment (**Figure 5A**); on blood agar, they were greyish white with a stimulating ammonia odour and a haemolytic ring (**Figure 5B**). The strain grew under conditions of 15–37 °C, pH 6–8, and NaCl concentration of 0–2%, but it did not grow below 4 °C and above 40 °C and did not tolerate 5% salt concentration like *S. maltophilia* and *S. terrae*. During semi-solid puncture culture, bacteria grew diffusely along the puncture line; however, no pigment was produced, indicating motility; pigment production was aerobic. 610A2^T^ was rod-shaped gram-negative, and without spores or capsules. Under electron microscopy, the bacterial body was about 1.5–2 × 0.5 μM with a single extreme flagella (**Figure 5C**).

**Figure 6:**
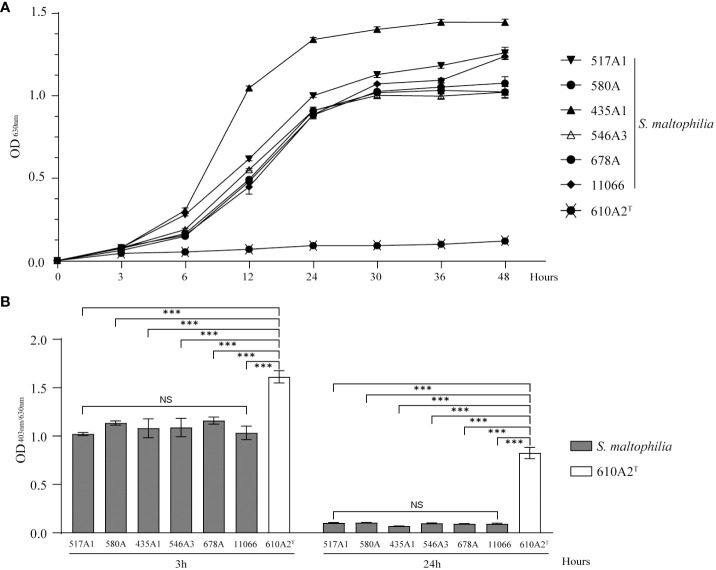
Growth curve **(A)** and biofilm formation **(B)** of 610A2^T^ and control strains in TSB medium. Significant differences between groups are marked with asterisks, ***P < 0.0002. NS, without statistical significance.

610A2^T^ grew slower than *S. maltophilia* strains in TSB. After 48 h, the cultures of 610A2^T^ showed turbidity, but the entire growth curve was flat without a logarithmic phase (**Figure 6A**). Although the growth rate was slow, the biofilm index showed that 610A2^T^ had a strong biofilm-forming ability, which was significantly higher than that of all *S. maltophilia* strains after 3 h of cultivation (**Figure 6B**).

The biochemical results showed that, in the API 20NE test, 610A2^T^ was positive for nitrate reduction, hydrolysis of quercetin and gelatin, assimilation of *N*-acetylglucosamine, maltose, and citric acid. In API ZYM, it was positive for alkaline phosphatase, esterase lipase (C8), leucine arylamidase, trypsin, acid phosphatase, and naphthol-AS-BI phosphate hydrolase. In API 50CH, only starch fermentation was detected. The major differentiating feature of 610A2^T^ was that it could not assimilate glucose and mannose, but it could ferment starch.

In the MALDI-TOF spectrum, the 4 main peaks of 610A2^T^ were located at 5254.065 Da, 4857.675 Da, 2770.713 Da, and 6118.187 Da, of which the 4857 Da peak was genus-specific and the 2770.713 Da peak was 610A2^T^-specific. In the local Bruker_MSP library, only 5 *Stenotrophomonas* species were found: *S. acidaminiphila*, *S. maltophilia*, *S. pictorum*, *S. rhizophila*, and *S. nitritireduce*ns. The identification scores of 610A2^T^ for these species ranged from 1.625 to 1.370, with the highest similarity to *S. pictorum*. These data indicate that 610A2^T^ is a distinct species closely associated with these species of *Stenotrophomonas*.

The predominant fatty acids of 610A2^T^ were iso-C_15:0_, iso-C_14:0_, summed Feature 3 (C_16:1_ω7c/_16:1_ω6c), iso-C_16:0_, and C_15:0_ anteiso and sum in Feature 9 (iso-C_17:1_ω9c). It contained all 3 fatty acids, iso-C_11:0_, iso-C_11:03_ 3OH, and iso-C_13:0_ 3OH, characteristic of the genus *Stenotrophomonas*, but no matches were found in RTSBA6. 610A2^T^ also contained all characteristic fatty acids of *S. terrae* and *S. humi*: iso-C_14:0_ (14.2% and 15.7%), iso-C_15:1_ (4.6% and 2%), iso-C_16:0_ (8% and 12.7%), and iso-C_17:1_ω9c (7.2% and 4.6%, respectively) (Heylen et al., 2007). The contents of these 4 fatty acids in 610A2^T^ were 15.45%, 1.5%, 11.9%, and 5.22%, respectively, indicating that the similarity of 610A2^T^ to *S. humi* is higher than that to *S. terrae*.

## 4 Discussion

When evaluating the pathogenicity of strain 610A2^T^, we used the outbred mouse KM, which has strong disease resistance and adaptability and can better reflect the genetic diversity of the human population. Within the first day post-infection, the bacterial load of 610A2^T^ in the lungs decreased sharply, and 610A2^T^ was cleared completely within 48 h. The lung clearance rate of 610A2^T^ in KM mice was faster than that of *S. maltophilia* in many inbred mouse strains, which also showed a larger decrease in CFU at 24 h, but could generally still be detected on day 3–7 post-infection and could even replicate in A/J mice with a transient increase in CFU at 4 and 8 h post-inoculation (Di Bonaventura et al., 2010; Rouf et al., 2011; Pompilio et al., 2018). After infection, both *S. maltophilia* (Di Bonaventura et al., 2010; Pompilio et al., 2018) and 610A2^T^ could be isolated in the spleen, suggesting that, at the same time of lung infection, *Stenotrophomonas* could disrupt the integrity of the lung epithelial barrier, cause bacteraemia, spread to other tissues, and pose an uncertain risk of assisting other pathogens in spreading and forming extrapulmonary lesions.

Combining with the acute infectious cytokine responses, the animal experiment in this study can confirm the pathogenicity of 610A2^T^ on the basis of the significant weight loss in the mice, tissue reactions due to inflammatory infection, and pulmonary congestion detected in the histopathological sections, although 610A2^T^ can be completely cleared from a healthy host. 610A2^T^ belonged to the *S. terrae* clade in the phylogenetic tree. In phylogenetic trees, whether constructed based on the concatenation of translated protein sequences (Patil et al., 2018) or the 16S rDNA gene, it is a clade that is relatively far from SMc. All species in this clade have been isolated from the environment and never been isolated from humans (Heylen et al., 2007). The isolation of 610A2^T^ changed the distribution of the pathogenic species. Together with *S. acidaminiphila* and *S. rhizophila*, all 3 main clades of the genus *Stenotrophomonas* have isolates from human sources.

610A2^T^ inherently possesses many pathogenic mechanisms, which can be inferred from the composition of functional genes on the genome, including those for motility (flagella and type IV pili), surface adherence (flagella, fimbriae, and LPS), damage capacity to host (protein secretion systems and extracellular enzymes), iron uptake and utilization, biofilm formation, protection against host defence (superoxide dismutase, hydroperoxidase, catalase, and melanin) as well as multi-antibiotic resistance (Di Bonaventura et al., 2010; Brooke, 2021). In addition, the genomic analysis suggested that 610A2^T^ produces type III haemolysin, for which erythrocyte lysis capacity and virulence have been confirmed in a mouse model infected with *Vibrio vulnificus* (Baida and Kuzmin, 1996; Chen et al., 2004; Moonah et al., 2014). In the genus *Stenotrophomonas*, colony pigmentation and brown diffusible pigments have been reported in only *S. maltophilia* (Romanenko et al., 2008; Liaw et al., 2010), *S. nitritireducens* (Finkmann et al., 2000), and *S. rhizophila* (Romanenko et al., 2008) isolates. Pigments and biofilm formation interact, and both are components of bacterial pathogenicity, enabling the colonisation or infection of hosts and promoting the attachment of other pathogens (Tamma et al., 2022). 610A2^T^ showed stronger abilities than *S. maltophilia* in both aspects, indicating that its pathogenicity may not be weaker than that of *S. maltophilia*.

However, the strain-specific cytokine responses induced by 610A2^T^ may be difference from those induced by *S. maltophilia*. In this study, 9 cytokines were selected on the basis of their inflammatory responses in *S. maltophilia-*infected murine model and patients with TB (Di Bonaventura et al., 2010; Mvubu et al., 2018). Among these cytokines, early inflammatory markers TNF-α and IL-6 showed consistently high expression after 610A2^T^ and *S. maltophilia* infection, with IFN-γ, IL-4, and IL-10 exhibiting different or even opposite changes (McDaniel et al., 2020) (**Table 2**). In mice infected with *S. maltophilia*, characteristic changes in cytokines were the persistent hyperexpression of IFN-γ for more than 3 days and sustained significantly lower level of IL-4 when compared with the control group (Di Bonaventura et al., 2010). IFN-γ was released in the early stage, along with large amounts of TNF-α and IL-2 and was closely associated with the ratio of T cells. Blocking the PD-1/PD-L1 pathway inhibited the apoptosis-inducing effect of *S. maltophilia* on T cells. The pleotropic cytokine IL-4 is a product of Th2 lymphocytes and inhibits Th1 cell differentiation. Therefore, the immune response to acute infection by *S. maltophilia* is predominantly a Th1-type response (Rouf et al., 2011). *S. maltophilia* may participate in the activation of T cells and induce subsequent T-cell exhaustion by activating the PD-1/PD-L1 signalling pathway, as the concentration of cytokines is reduced in the later stages (Wang et al., 2021; Xu et al., 2023). In contrast, in the 610A2^T^-infected KM mice, cytokines IL-4, IL-6, and IL-10 secreted by Th2 cells showed a rapid and significant increase; there was no difference in IL-12, the main cytokine that induces Th1 cell differentiation and inhibits Th2 cells, indicating that the immune response of 610A2^T^ was Th2 type (**Figure 4**). In addition, the lagging release of IFN-γ and IL-2 in 610A2^T^-infection indicated that Th1 type was disinhibition in the later stage and also involved in the host’s defence against the pathogen. In-depth research of the pathogenic *Stenotrophomonas* species including 610A2^T^ in same mouse strain infection model will further elucidate pathogenic and immunological characteristics.

**Table 2:** The inflammatory responses of *S. maltophilia* and 610A2^T^ infected mice and TB patient.

Cytokines	Classification	*S. maltophilia*		610A2^T^	TB patient^3^
		A/J mice ^1^	DBA/2 mice^2^	KM mice	
TNF-α	Pro-	*↑	*↑	*↑	*↑
IFN-γ	Pro-	*↑	*↑	*↑	*↑
GM-CSF	Adaptive	–	–	*↑	*↑
IL-1β	Pro-	–	*↑	–	ns
IL-2	Adaptive	–	ns↓↑	*↑	*↑
IL-4	Adaptive	–	*↓	*↑	*↑
IL-5	Adaptive	–	ns↑↓	–	ns
IL-6	Pro-	*↑	*↑	*↑	*↑
IL-10	Anti-	–	ns↓↑	*↑	*↑
IL-12	Anti-	–	*↑	ns	*↑
IL-17A	Pro-	–	–	*↑	*↑

^1^ Data from (Rouf et al., 2011); ^2^ Data from (Di Bonaventura et al., 2010); ^3^ Data from (Mvubu et al., 2018). * cytokines having significantly different between infected and control mice; ns without statistical significance; – no included in study. ↑ the concentration of cytokine in the infection group is higher than that in the control group; ↓↑ during the observation period, the concentration of factors in the infection group is initially lower than that in the control group, and then increase to a level higher than that in the control group.

Moreover, 610A2^T^ is resistant to half of the commonly used anti-TB drugs, including isoniazid, and carries ESBL and metallo-β-lactamase, which can hydrolyse all bicyclic β-lactam antibiotics (Hinchliffe et al., 2023). The co-existence of *Stenotrophomonas* may affect therapeutic efficacy in patients with TB. The AST results showed that all drugs recommended by the Infectious Diseases Society of America for the treatment of *S. maltophilia* infection (Tamma et al., 2022) may be applicable to 610A2^T^, except TMP-SMX, which is recommended as the first-line agent for the treatment of *S. maltophilia* infection owing to the low isolation rate of resistant strains (Chang et al., 2015; Gröschel et al., 2020). A combination of colistin and rifampicin may be used for patients with mixed 610A2^T^ and TB infections.

### 4.1 Description of *Stenotrophomonas pigmentata* sp. nov.

*Stenotrophomonas pigmentata* sp. nov. (pig.men.ta’ta. L. fem. adj. *pigmentata*, pigmented, coloured).

The type strain 610A2^T^ was isolated from patients with pulmonary TB. It is a Gram-negative bacillus with a single extreme flagella and can produce brown water-soluble pigment. Growth is observed at 15–37 °C, pH 6–8, and 0–2% NaCl concentration, but it does not grow below 4 °C, above 40 °C, and at 5% salt concentration. 610A2^T^ yielded positive results for nitrate reduction, quercetin and gelatin hydrolysis, and starch fermentation. The 4 main peaks in the MALDI-TOF spectrum were located at 5254.065 Da, 4857.675 Da, 2770.713, Da and 6118.187 Da. The predominant fatty acids are iso-C_15:0_, iso-C_14:0_, summed Feature 3 (C_16:1_ω7c/_16:1_ω6c), iso-C_16:0_, and C_15:0_ anteiso and sum in Feature 9 (iso-C_17:1_ω9c). 610A2^T^ has multi-drug resistance and intrinsic resistance to β-lactams, carbapenems, and trimethoprim-sulfamethoxazole, harbouring several multi-drug resistance efflux pump and antibiotic resistant genes. This strain is pathogenic to mice.

The complete genome of type strain 610A2^T^ comprises 4681496 bp and GC content of 63.29%, and the complete genome and 16S rDNA gene sequence are deposited in GenBank under accession numbers CP130832.1 and OR936313, respectively. The type strain is 610A2^T^ (=GDMCC 1.4134 ^T^ =JCM 36488 ^T^).

## Data availability statement

The datasets presented in this study can be found in online repositories. The names of the repository/repositories and accession number(s) can be found below: https://www.ncbi.nlm.nih.gov/genbank/, CP130832.1, https://www.ncbi.nlm.nih.gov/genbank/, OR936313.

## Ethics statement

The animal study was approved by the Laboratory Animal Welfare & Ethics Committee of the National Institute for Communicable Disease Control and Prevention (Issue number 2023-021). The study was conducted in accordance with the local legislation and institutional requirements.

## Author contributions

YL: Investigation, Writing – review & editing. ZY: Investigation, Writing – review & editing. XF: Investigation, Writing – review & editing. DX: Investigation, Writing – review & editing. HL: Writing – review & editing. XZ: Investigation, Writing – review & editing. RW: Conceptualization, Data curation, Formal analysis, Investigation, Methodology, Project administration, Resources, Supervision, Visualization, Writing – original draft, Writing – review & editing.
